# Reduced graphene oxide (rGO) based wideband optical sensor and the role of Temperature, Defect States and Quantum Efficiency

**DOI:** 10.1038/s41598-018-21686-2

**Published:** 2018-02-23

**Authors:** Abid, Poonam Sehrawat, S. S. Islam, Prabhash Mishra, Shahab Ahmad

**Affiliations:** Centre for Nanoscience and Nanotechnology, Jamia Millia Islamia (A Central University), New Delhi, 110025 India

## Abstract

We report a facile and cost-effective approach to develop self-standing reduced Graphene Oxide (rGO) film based optical sensor and its low-temperature performance analysis where midgap defect states play a key role in tuning the crucial sensor parameters. Graphite oxide (GO) is produced by modified Hummers’ method and reduced thermally at 250 °C for 1 h in Argon atmosphere to obtain rGO. Self-standing rGO film is prepared via vacuum filtration. The developed film is characterized by HRTEM, FESEM, Raman, and XRD techniques. The developed sensor exhibits highest sensitivity towards 635 nm illumination wavelength, irrespective of the operating temperature. For a given excitation wavelength, photoresponse study at low temperature (123K–303K) reveals inverse relationship between sensitivity and operating temperature. Highest sensitivity of 49.2% is obtained at 123 K for 635 nm laser at power density of 1.4 mW/mm^2^. Unlike sensitivity, response- and recovery-time demonstrate directly proportional dependence with operating temperature. Power dependent studies establish linear relation between power-density and sensitivity, and a safe limit beyond which sample heating prolongs the recovery time. Wavelength-dependent studies shows that proposed sensor can efficiently operate from visible to near NIR region. To the best of our knowledge such rGO based optical sensor performance at low temperature had not been reported earlier.

## Introduction

Photodetectors having superior detection parameters such as sensitivity, speed of response, internal as well as external quantum efficiency, detection bandwidth and flexibility are in great demand for their widespread applications in sensing, imaging, etc.^1–3^. In this regard, apart from the choice of material, dimensionality also significantly affects the sensor performance. Recent advancements in the frontiers of nanotechnology have opened new avenue and pathways for researchers to look for novel materials for a variety of applications. Graphene has turned out to be one of the most celebrated inventions in post silicon era because of its extraordinary and interesting properties: small thickness (of the order of one atomic layer), large surface to volume ratio, very low mass, high mobility of charge carriers, and decent absorption coefficient, etc.^4–8^.

Graphene is a semi-metallic material because of its special π-π* band structure. At Dirac point (charge neutrality point) the conduction and valence bands are symmetrical. The superior electrical and optical characteristics in graphene are because of linear dispersion of Dirac massless electrons moving with a fraction of the speed of light. In pure graphene, fermi energy lies at charge neutrality point, so its electronic properties near *k*-point (Dirac point), the conductance is expected to be a minimum. Under external stimulus, the entire band structure can be shifted with respect to the fermi level and pave the way to induce enhanced conductivity via: (a) increasing the magnitude of gate voltage (in FET)^9–11^, (b) excess electron-hole pair generation by irradiating light (in photoconductor)^12^, and (c) enhanced electron-phonon scattering (in thermal conductivity) on either side of the Dirac point^9,11^.

A good photodetector should detect light of a wide spectral range. Graphene’s capacity of absorbing ~2.3% of incident light and its high mobility renders the realization of broadband photodetector with ultrafast detection (>500 GHz)^13,14^. On the contrary, its poor photo-responsivity^14^ and extremely short carrier lifetime (of the order of picoseconds)^3^, which originates from its intrinsic zero-band gap energy, does not allow graphene to be the best candidate for photodetection. One way to solve this issue is bandgap opening^2,14^. Defect engineering is a versatile option through which mid-gap states can be created within the band structure of graphene to resolve two prominent issues simultaneously: (a) to slow down the carrier recombination time by charge trapping, and (b) increase in bandgap also increases the absorption efficiency compare to pristine graphene^14^. An easy way to achieve this is to transform graphene oxide (GO) to reduced graphene oxide (rGO). The band gap in GO is ~2.2 eV and for rGO, bandgap can vary from ~1.00 to 1.69 eV depending on the degree of reduction^15–19^. On reduction, some of the oxygen groups are removed and bandgap can therefore be adjusted further by managing the oxygen present in rGO. Thus, reduced graphene oxide (rGO) behaves like semi-metal or semiconductor and its electrical conductivity may be tuned by controlling its oxygen content^16^.

In 2014, Haifeng Liang has reported mid-IR photoresponse of rGO with a responsivity of 1 A/W^17^. Very recently, pristine graphene based infrared photodetectors are demonstrated with an optical modulation of up to 40 GHz, however a low responsivity (~6.1 mA/W), caused by rapid photocarrier dynamics, is compromised^18^. In 2013, Haixin *et al*. reported photoresponse of rGO based phototransistor operated in infrared region by controlling the defects and atomic structure; remarkable infrared photoresponse of ~0.7 A/W (at 895 nm) and external quantum efficiencies (EQE) of 97% is reported^19^. D. Sun *et al*. reported ballistic photocurrent generation in a graphene based photodetector^20^. Notwithstanding the gapless nature of graphene, strong photocurrent response is reported at metal/graphene interface having internal quantum efficiency ~15–30%^21^. Chang-Hua Liu *et al*. designed a wide-broadband photodetector using graphene bi-layer heterostructure^22^. In 2017, Jing Jing *et al*. have reported ultra-high responsivity of upto 10^5^ A/W at room temperature in silicon-graphene conductive photodetector^23^. All these disparate responses are found due to varying types of graphene synthesis routes, methodology, device engineering and fabrication.

Sensor design by placing a monolayer graphene between the electrodes in lateral configuration is difficult and an expensive task, since it requires high-end nanoscale manipulation techniques^24^. Other disadvantage is its less absorption area, which in turn limits the responsivity of sensor. Hence, to enhance the photo-response, we need to focus on three prominent factors – (a) design a sensor that provides sufficient absorption area, (b) convert the precursor graphite powder to rGO to enable maximum light absorption for generation of e-h pairs in excess, and (c) drift the generated carriers towards electrodes to avoid band to band recombination, otherwise ultrafast (~*ps*) recombination of e-h pairs will fail the prospects of the sensor due to its zero-bandgap nature. On the flip side, we sacrifice the speed of the device because defects slow down the mobility of charge carriers and narrow down the detection bandwidth^3^. Researchers have been trying to trade-off this dilemma by considering several aspects including material synthesis, device design and fabrication strategies. Even efforts were made to develop rGO mesh/network structure by spray deposition as well as synthesizing composite with polymers, glues, epoxies, etc.

In this paper, we report the temperature dependent photo-sensing response of rGO based photo-detector. The free-standing rGO film is developed by vacuum filtration technique^25,26^. The processed film is a mesh of well dispersed graphene sheets with an average size of ~2 μm. The device performance has been studied against various sensor parameters such as excitation wavelength (visible to NIR), illumination power density and low temperature. Unlike sensitivity, response- and recovery-time demonstrate directly proportional dependence with operating temperature. Highest sensitivity of 49.2% is achieved at 123 K for 635 nm wavelength at 1.4 mW/mm^2^ laser power density, indicating the potential of the sensor to operate even in cryogenic conditions. Power dependent photoresponse indicate linear variation of sensitivity with power density upto certain extent beyond which bolometeric effect comes into action and extends the sensor recovery time. The sensor exhibits high sensitivity for wide range of illumination from Vis to NIR signifying its potential device applications for wide bandgap photodetectors.

## Results and Discussion

### Graphene (GO) to reduced Graphene Oxide (rGO): Preparation

Graphene oxide (GO) is produced from graphite powder using modified Hummers’ technique^27^: A round bottom flask is kept in an ice bath and filled with 120.0 mL H_2_SO_4_ (95%). Graphite powder (5.0 g) and NaNO_3_ (2.5 g) are added to the flask under vigorous stirring. Followed by this step, 15.0 g KMnO_4_ is mixed under continued stirring at a temperature of less than 10 °C. After mixing KMnO_4_, ice bath is taken off and solution is stirred at 30 °C for a day. The mixture progressively grew thick (paste) and turned light brown in color. Following this, 150.0 mL of deionized water is gradually added in this mixture under constant stirring. The diluted suspension is stirred for 2 h at 98 °C. Subsequently, the temperature is decreased to 60 °C, and 50.0 mL H_2_O_2_ (30%) is further added in the mixture in order to remove any leftover MnO^−^_4_. Lastly, the resultant mixture is filtered and rinsed with deionized water to obtain neutral pH. The extracted mixture is then desiccated at 80 °C and graphite oxide is obtained as powder which is exfoliated to GO sheets using a ultrasonication (1hr, 500 W, 40 kHz) followed by centrifugation (3000 rpm) to separate unexfoliated graphite oxide.

Ultrasonication time is varied (1 h, 3 h, and 5 h) in order to obtain monolayer GO sheets. This was followed by vacuum filtration by using cellulose nitrate filter membranes (pore size: 0.22 μm; diamater: 47 mm). Further, the film is desiccated at 60 °C in a hot air oven for 24 h and peeled off from the filter as self-standing GO film. Finally, this film is thermally reduced at different temperatures (100 °C, 150 °C, 200 °C, and 250 °C) in inert atmosphere for different annealing times (10 min, 30 min, 60 min, 180 min, and 300 min) to optimize the film quality to achieve best performance.

**Figure 1:**
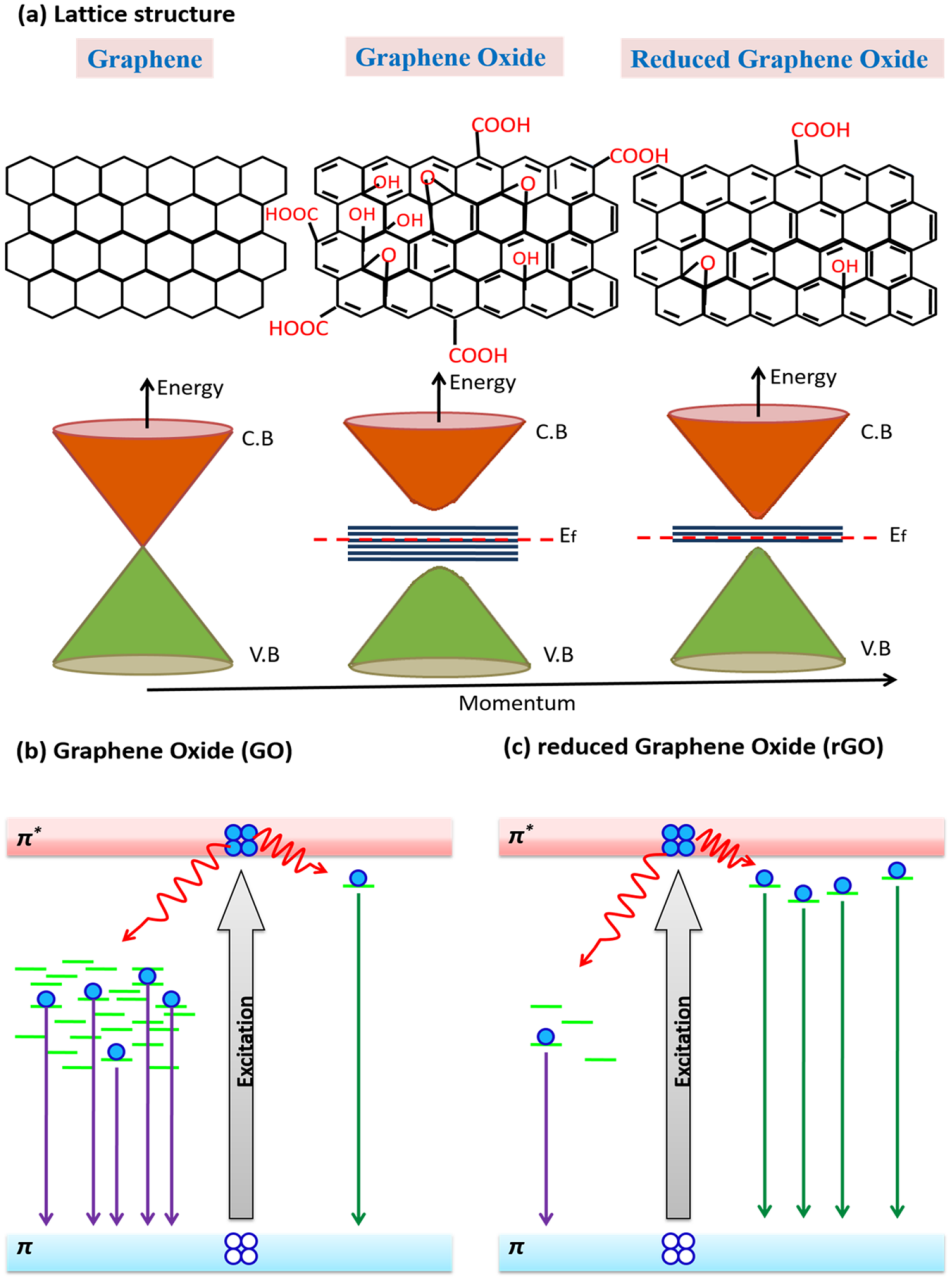
Schematic representation of: (**a**) Lattice structure and corresponding energy band diagrams of Graphene, GO and rGO; and electronic transitions in (**b**) GO and (**c**) rGO.

The understanding of lattice as well as electronic band structure of graphene, GO and rGO is necessary to understand how the transformation of GO to rGO leads to high photoexcitation response. The atomic structure of GO is purported as a graphene basal plane having inconsistently dispersed oxygen-containing functional groups (hydroxy and epoxy units) resulting in *sp*^3^ matrix (Fig. 1(a)) which makes it an electronic insulator^28^. Figure 1(b,c) show electronic band structure of GO and rGO respectively. The ordered hexagonal lattice of graphene becomes disordered by oxygenous defects produced during chemical oxidization process. The optoelectronic properties of carbon materials having a combination of *sp*^2^ and *sp*^3^ bonding are primarily controlled by *π* and π* states of *sp*^2^ locations, lying in σ–σ* gap^29,30^. Since *π* bonding is weaker with low formation energy, it is possible that numerous disorder-induced localized states may appear within the two-dimensional (2D) network of as produced GO^29^. The structural disorder-induced localized states might be present in the band tail of π–π* gap or lie deep inside this gap. Accordingly, optical transitions involving these disorder-induced localized states may cause wide absorption or emission bands^29,30^.

The quantity of disorder-induced states declines after reduction owing to deoxygenation. This structural modification is attributed to the reduction of oxygenous functional groups and gradual recovery of the conjugations between carbon atoms during reduction process^3,29^. As a result, certain carbon lattices from the initial distorted *sp*^2^ domains may create additional graphitic domains of *sp*^2^ clusters^3,29^. These small *sp*^2^ clusters, create isolated molecular states, finally percolate to facilitate the charge carrier transport by hopping^28^. The reduction process leads to increased carrier mobility, higher absorption, a tunable bandgap where photoresponsivity can be controlled by controlling the defects and oxygen groups^3,19,29^.

## Material Characterization

### Morphology studies

Surface morphology of the self-standing film prepared by vacuum filtration is thoroughly examined by Field Emission-Scanning Electron Microscope (∑igma, Zeiss). The SEM image of film is uniform with little wrinkles as evident in the SEM micrographs shown in Supplementary Information (Figure S2(a,b) and Figure S2(c,d)) provides the cross-section view of fabricated self-standing film. Figure S2(d), also illustrate the stacking of the graphene oxide.

**Figure 2:**
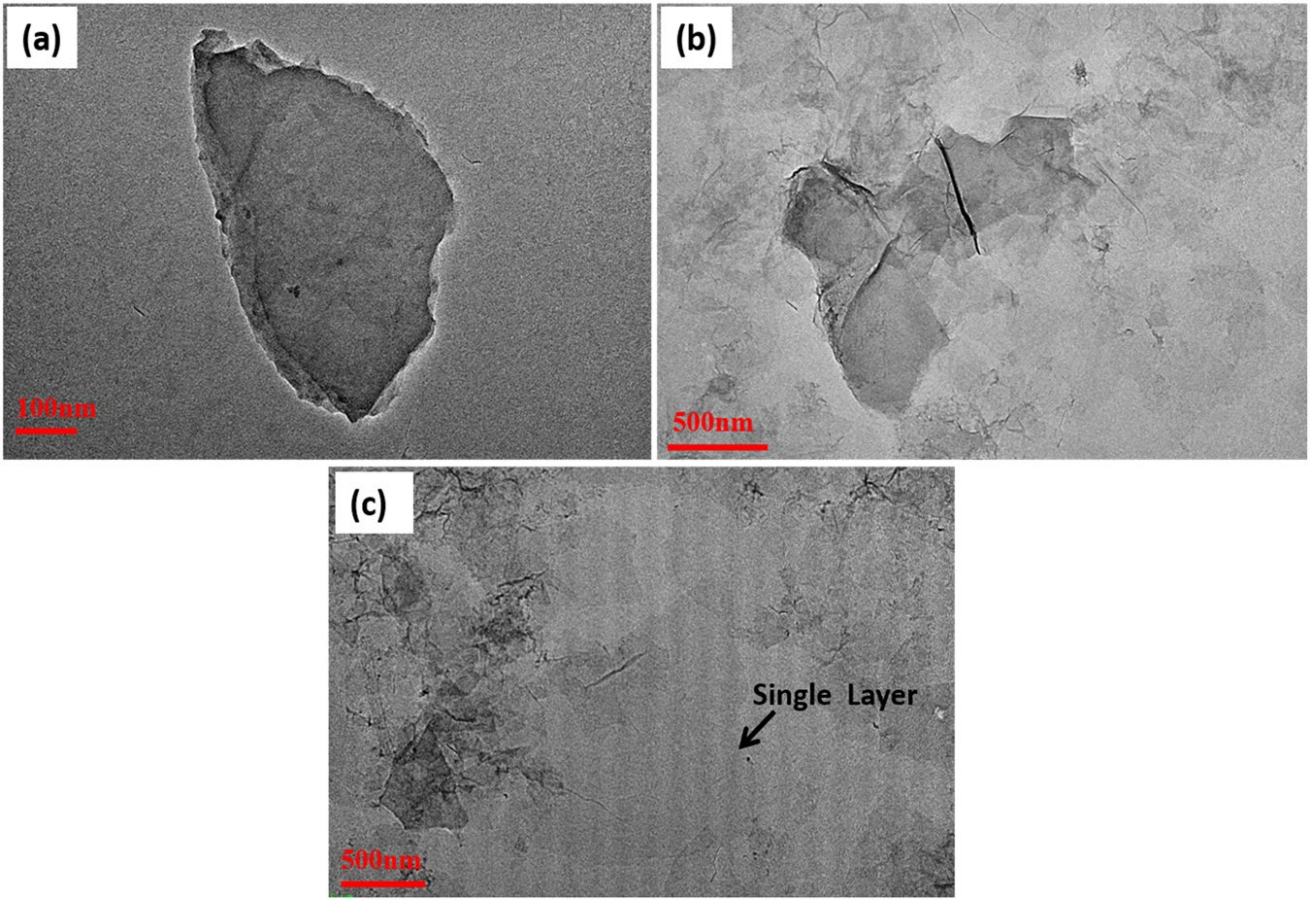
HRTEM images of GO as a function of ultrasonication time (**a**) 1 h, (**b**) 3 h, and (**c**) 5 h.

Figure 2(a–c) shows High Resolution-Transmission Electron Microscope (Technai G^2^ 30 S Twin) images of GO sheets that are highly transparent with some overlaps and wrinkles. From figure it is clearly visible that as ultrasonication time is increased (ranging from 1 h, 3 h, to 5 h), the transparency of the sheets has enhanced significantly and GO sheets can be exfoliated to single layer by increasing the ultrasonication time. We have produced GO that has single as well as few layer flakes/ribbons with an average size of ~2 µm.

### Characterization of defects by Raman spectroscopy

**Figure 3:**
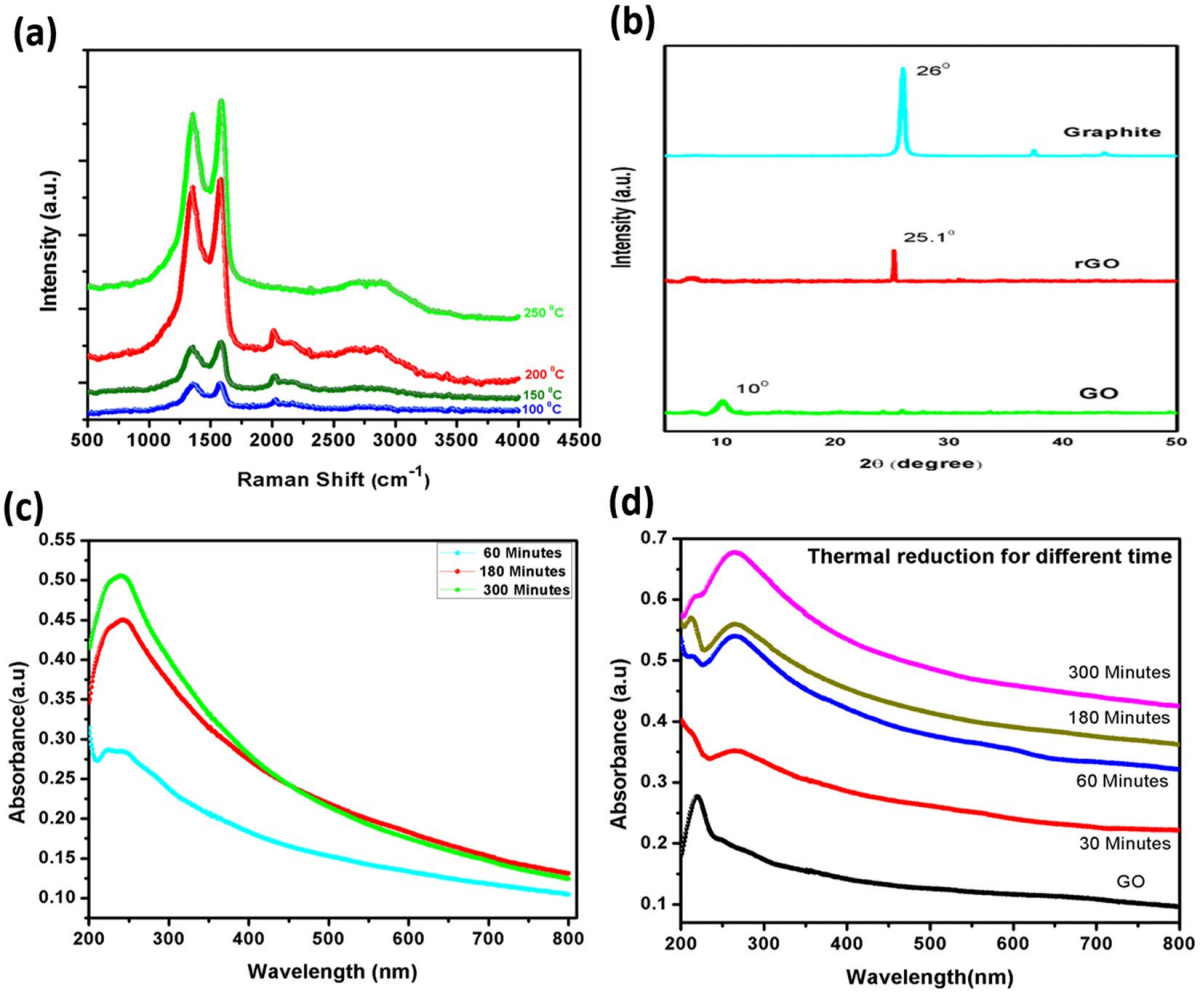
(**a**) Raman spectra of reduced graphene oxide (rGO) film at different thermal reduction temperature. (**b**) XRD patterns of graphite, GO, and rGO films. UV-Vis spectra of (**c**) GO dispersion as a function of ultrasonication time, and (**d**) rGO film as a function of thermal reduction time. Thermal reduction is performed at 250 °C.

Raman characterization is an extensively employed technique to get the structural information of the material. Characteristic peaks in the Raman spectra of graphene are designated as D (disorder), G (graphitic), and 2D. The first order D and G peaks appear at ~1350 cm^−1^ and 1580.4 cm^−1^, arising due to vibrations of *sp*^2^ carbons. The G- and D-peak intensities are important to measure the graphitic character and the degree of disorders respectively. In GO, large band width is indicative of large structural disorders. The 2D peak occurs at 2706 cm^−1^ in GO and rGO and intensity is characteristically weak as compared to pristine graphene. Figure 3(a) shows the Raman spectra of film obtained after vacuum filtration and reduced at different temperature. In this figure it is easily understood that as the temperature increases, 2D peak starts appearing.

### XRD-spectroscopy

Figure 3(b) includes XRD patterns of graphite, GO and rGO (annealed at 250 °C for 1 h). As shown, graphite flakes exhibit a strong and sharp diffraction peak at 26°, which corresponds to the well-ordered layered structure of graphite. Graphite is treated under strong chemical oxidation process to produce exfoliated GO, where oxygen functional- and epoxy groups are introduced in between the consecutive layers due to which diffraction peak shifts to lower diffraction angle (2*θ* = 10°). The rGO sample shows a new strong peak at 2*θ* = 25.1° and it is attributable to the removal of oxygen containing functional groups, resulting in reduced *d*-spacing compare to GO.

### UV-Vis spectroscopy

Figure 3(c) provides the UV-VIS spectra of GO where dispersion is achieved by ultrasonication for 1 h, 3 h, and 5 h. Maximum absorption peak at ~237 nm is observed, resulting from π-π* transition of aromatic C-C bonds. Absorption peak for rGO is red shifted to 266 nm, and this shift is considered as a measure for the reduction of GO^31^. Films obtained from vacuum filtration method is thermally reduced at various temperature (100 °C, 150 °C, 200 °C, and 250 °C) in inert atmosphere for different times (10 min, 20 min, 30 min, 60 min, 180 min, and 300 min). Figure 3(d) show the UV-Vis spectra of rGO sample reduced at 250 °C for different times.

### Fabrication of resistive optical sensors

**Figure 4:**
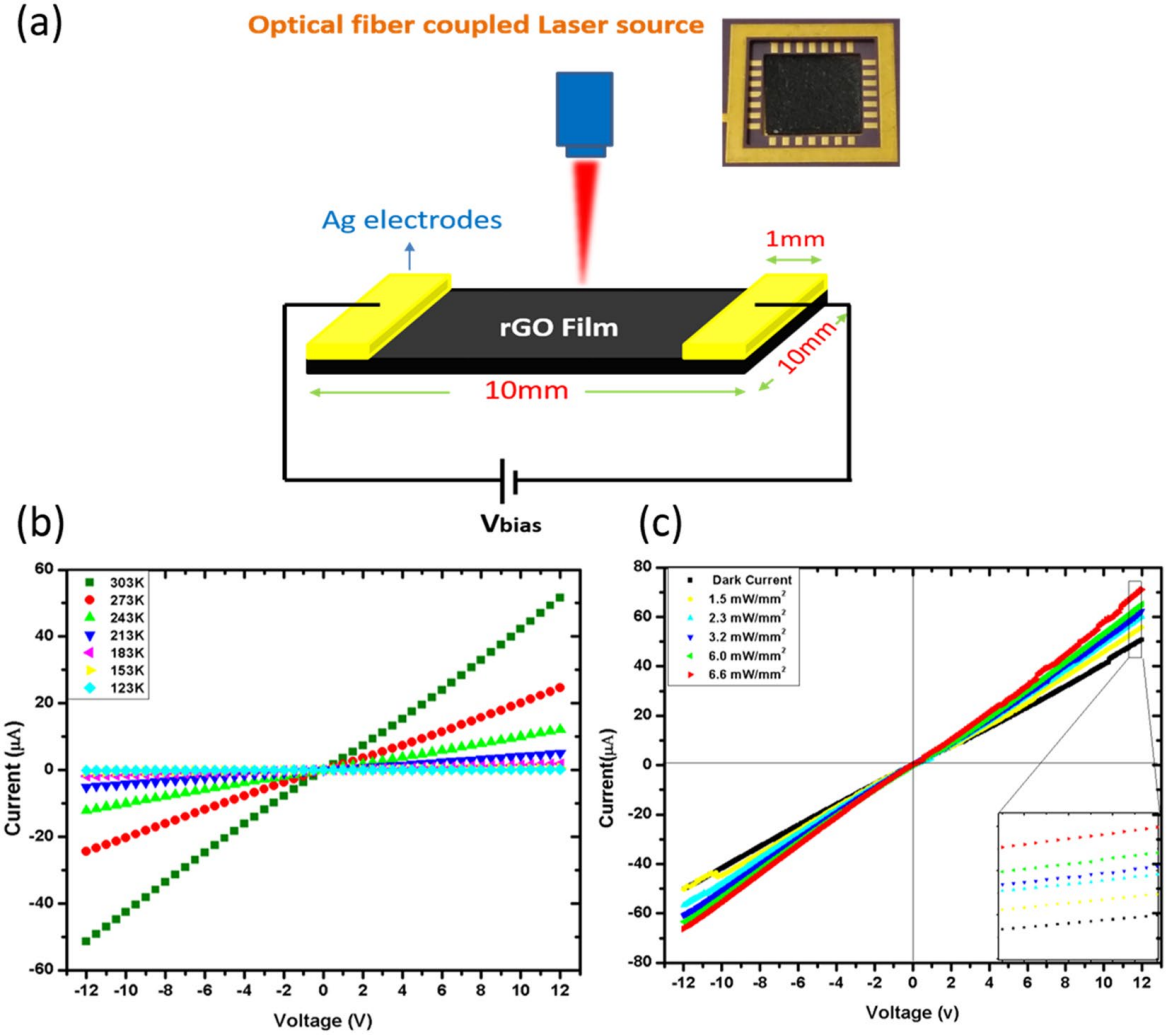
(**a**) Schematics of the optical sensing set up and inset shows the photograph image of the fabricated optical sensor. Current-voltage characteristics measured at different (**b**) temperature and (**c**) laser power density.

Optical sensors were developed from the self-standing rGO film by cutting 1 cm × 1 cm pieces and depositing silver electrodes on both ends. Figure 4(a) shows the schematic diagram and photograph of the prepared sensor along with physical dimensions. The electrodes were left to dry for 24 h in ambient atmosphere. Figure 4(b,c) shows current versus voltage characteristics measured at different temperature and laser power density. Metal/semiconductor (M/S) contact shows ohmic behavior due to quantum tunneling effect^32^. The fabricated rGO film in this device configuration works as a photosensitive sensor and its conductance increases upon light illumination.

The sensor was placed in the sample chamber (Linkam, UK), and the electrical signal is measured with Keithley SCS 4200 system. The light from diode laser source is irradiated on the sensor from top side, as shown in Fig. 4a, with the help of three different lasers (BWF1, B&W Tek) of λ_exc_ ~ 635 nm, 785 nm, and 1064 nm of tunable powers. The distance between laser source and sample was kept constant at 10 mm with a spot size of approximately 6 mm comfortably confined within the sensing area. The laser power was recorded using a Newport 843 R power meter.

### Generation and recombination of photocarriers under photoexcitation and bias field

As recognized, transformation of GO to rGO makes rGO flakes which is nothing but stacking of graphene-like layers. For layer based photodetectors, even applicable to rGO, three types of main photocurrent generation mechanisms are proposed: photoconductive effect, photovoltaic effect, and photothermoelectric effect^2,9,33–35^. We can exclude the photogenerated thermoelectric effect for photocurrent, because a homogeneous temperature field can be achieved in the device by focusing light to cover the entire device and the photothermal currents flowing in two different directions are cancelled out on contacts^36^. This is evident in our sensor recovery plot as evident in the studies carried out in the section Temperature dependent Sensing Response. Any heating effect makes a slow exponential decay^22,37,38^ and this is absent in our case. Moreover, as evident from I-V characterization shown in Fig. 4(a and b), there is no short-circuit photocurrent (J*sc*), therefore, the photovoltaic effect may be ruled out. These results strongly suggest that the photoconductive effect is the dominant mechanism of photocurrent generation in the rGO based photodetector^33^.

The general operational principle of a solid-state photodetector involves: (a) generation of carriers by absorbing incident photons within the semiconducting layer, (b) transport and multiplication (if available) of these carriers, and lastly (c) driving out these carriers into the external circuit to circulate until the photogenerated carriers (e^−^ and h) recombine together^39^. While drifting within the channel space, photogenerated carriers may get confined to trap/defect states present within the bandgap as well as at the rGO/electrode interface, and subsequently de-trap by incident photons or thermal vibrational energies. Therefore, under light illumination, confined carriers tend to return to their respective bands instead of recombining; this is because- (a) the incident photons have much higher activation energy than the trap barrier potential, and (b) photon flux is sufficient to reduce the effective recombination rate of carriers^40^.

At any temperature, the principle of detailed balance between generation and recombination may be worked out considering the interband as well as Auger recombination processes in case of graphene and related materials. A detailed analysis is available in the literature and readers may refer to the references therein^3,39–42^. For traps assisted recombination, there are two dominant mechanisms for graphene layers. One is the Shockley-Read-Hall recombination (*R*_*SRH*_)^39–43^ and the other is the trap involving Auger- recombination. (*R*_*Auger*_)^43–47^. In the limit of high carrier density, these can be simply expressed as^35^:

1 $${{\rm{R}}}_{{\rm{SRH}}}=\frac{{{\rm{\alpha }}}_{e}{{\rm{\alpha }}}_{h}{V}_{th}{N}_{t}{r}^{2}{n}_{e}{n}_{h}}{({n}_{e}{\propto }_{e}+{\alpha }_{h}{n}_{h})d}\cong \frac{{\alpha }_{e}{V}_{th}{N}_{t}}{d}r{n}_{e}$$

2 $${{\rm{R}}}_{{\rm{Auger}}}={C}_{Auger}{({{\rm{rn}}}_{{\rm{e}}})}^{2}\propto \frac{{N}_{t}{({{\rm{rn}}}_{{\rm{e}}})}^{2}}{d}$$

Here *α*_*e(h)*_ represents the capture cross sections to trap electrons (holes), and *N*_t_ is the areal density of traps. Under illumination, the exchange of electrons mainly takes place between bands and trap/defect states; therefore, any variation in number of electrons either in the conduction band or in trap/defect states must include all possible processes that take part such as -

- Change in the number of conduction band electrons occurring due to photogeneration, carrier multiplication (carrier impact ionization), recombination and relaxation to the defect states, and
- Change in the quantity of electrons on the defect states occurring due to excitation to the conduction band, relaxation from conduction band, and recombination with holes in the valence band.

Considering these facts, the electron capture rate (*α*) of the trap/defect states as described by Yongzee Zhang *et al*.^3^ is

3 $$\alpha =\frac{1}{e\chi \beta }({\tau }_{t}R/{\tau }_{1})$$

and the quantum efficiency (*η*) of the photoexcited and secondary-generated electrons trapped by defect states is written as^3^,

4 $${n}=(\frac{I}{e\chi \beta {\tau }_{1}}){\tau }_{t}$$

where ${\tau }_{1}$ and ${\tau }_{t}$ are the lifetime of trapped electrons and transition time respectively. *χ* is the number of electrons per absorbed photon due to electron and hole impact ionization, and *β* represents photogeneration rate.

From Eqs (3) and (4),

5 $$n=\frac{I\alpha }{R}$$

where *R* is the electron recombination rate.

In Eq. (5), both the terms *α* and *R* are temperature dependent. In case of graphene, *R* ~ 1 *ps*^43^ and assumed to remain unchanged due to negligible change in its order at higher temperature. Therefore, *α*, the electron capture cross-section rate by the defect states, will be the ultimate decisive factor to control *η*; In a nut shell, photoresponsivity (*R*_*λ*_) as well as the external quantum efficiency (EQE) (refer to Supplementary information file) of the sensor device is the outcome of both the factors *α* and *η* for low and high temperature of operations.

### Electron Scattering Mechanism

There are many defects or trapping states in multilayered rGO flakes that influence free charge carrier density in the conduction band. It is supported by the linear dependence of photoconductivity on light power density^44^ as evident in the studies carried out in the section Temperature dependent Sensing Response. Photogenerated electrons can be trapped in the defect states and remain there at temperature not sufficient to overcome the capture probability of the defect states. Owing to charge conservation in the carrier conduction channel, multiple hole-circulation occurs after a single photon generates an e-h pair, and the holes do not recombine with electrons until the dilution of the said capture probability^45,46^.

Thus, the factors that lead to the generation and the significant loss in the extraction/collection of photogenerated charge carriers may be summarized as follows:

- Decrease in electron capture probability with the defect states leading to release/generation of free carriers to the conduction band. This corresponds to the transition of electrons from defect energy state (lying below conduction band) to conduction band.
- Electron-hole pair non-radiative recombination through electron transition from conduction- to valence band leading to loss of electron and hole together in their respective bands.
  The factors that delay the recombination process as well as increase the electron transit time (${\tau }_{tr})$ to reach to the terminal electrode are^47^.
- Electron- phonon scattering
- Electron-electron scattering
- Electron- boundary scattering
- Electron- defects scattering
- Electron –interface or grain boundary scattering.

Factors (a) i.e. the electron capture probability is concerned with the defect energy states confined in the forbidden gap, and it is inversely proportional to the temperature of operation^46^. It means that at high temperature, less number of electrons will remain captured or trapped with the defect states and therefore, there will be generation of excess free carriers available in the conduction band of rGO; and it is vice versa for low temperatures. At every higher temperature, the thermal equilibrium carrier density will be higher and different vis-à-vis the previous lower temperature^39^. The thermal equilibrium carrier density at temperature T is, in general, of the order 10 ^*m*^. On light illumination, the carrier density further increases by a factor say 10^*n*^ resulting in the net free carrier density (in the conduction band) of the order 10^*m*+*n*^, where *m* and *n* are the scaling exponent factor and their values are >>1^39^.

The second factor (b) i.e. the ‘e-h pair non-radiative recombination’ should theoretically reduce the number of carriers but this is prevented to a very large extent by applying the optimized bias voltage (V_*bias*_) across the sensor device to drift the carriers and this is how the photoconductive detector works. In addition, this recombination loss probability is further reduced due to the random scattering factors (c-g). Higher the temperature, higher the scattering, and lesser is the recombination probability. As the scattering factors increase, the carrier transit time (${\tau }_{tr}$) increases by a factor 10^*p*^, (where *p* ~ 1), leading to a decrease in collection of net charge carriers (*Q*) at the terminal electrode^48^; this will reduce the terminal current *I* (*I*_*terminal*_ = *Q*/${\tau }_{tr}$), subsequently decrease the sensitivity with rise in temperature as seen in the studies of the section Temperature dependent Sensing Response. Some fraction of carrier loss cannot be ruled out from quantum mechanical point of view but very less in comparison to carrier generation factor^44^. Considering these facts, the photocurrent as well as external quantum efficiency (EQE) of the sensor device should increase with increase in temperature due to the dominance of carrier generation factor over the delay in transit time. Temperature dependent experimental results endorse these facts and suitable analysis is given in the next sections.

**Figure 5:**
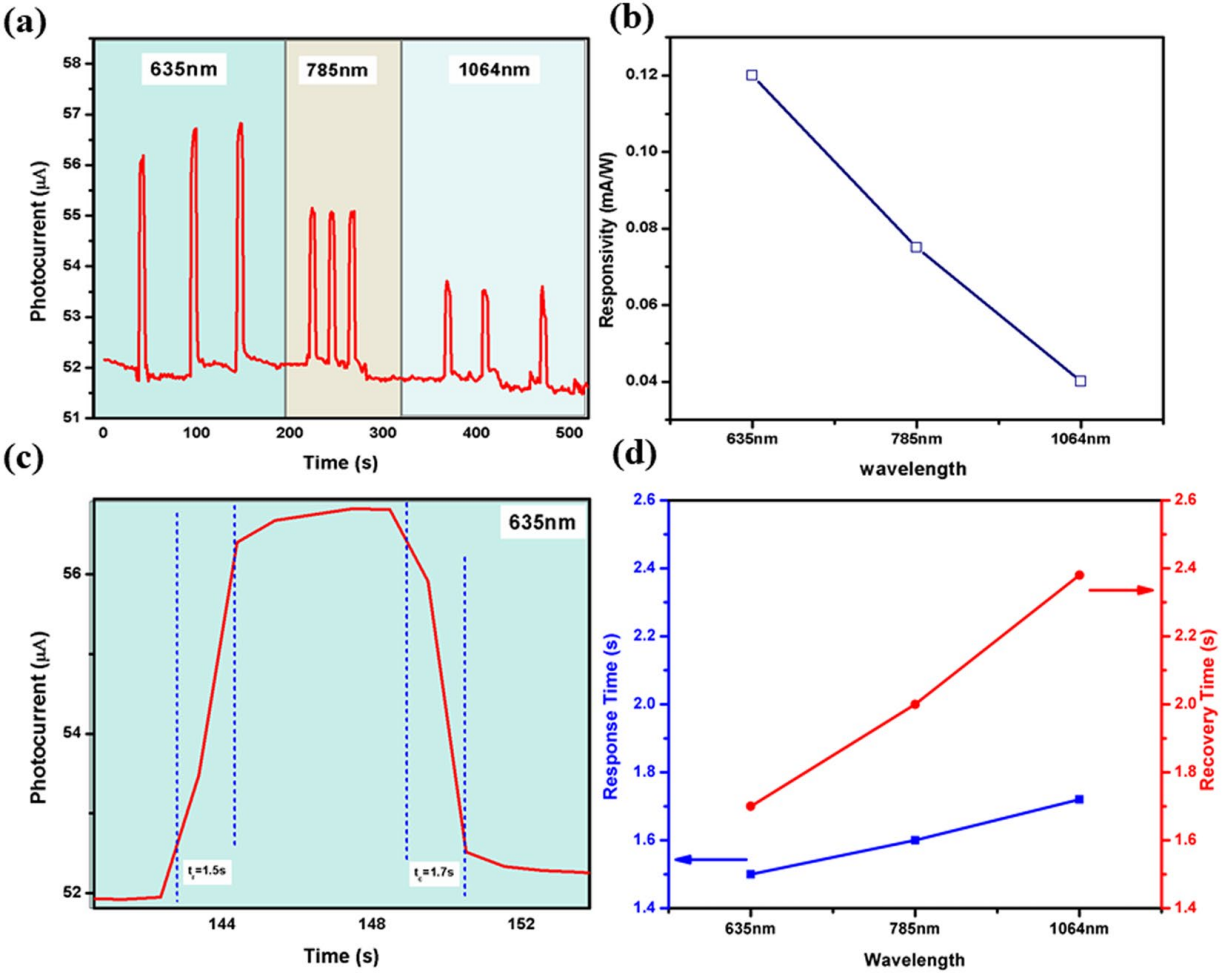
(**a**) The photocurrent vs time plot of rGO sensor for various excitation wavelengths: 635 nm, 785 nm, and 1064 nm (**b**) corresponding photoresponsivity of sensor illuminated under different wavelengths, (**c**) response and recovery time of sensor when illuminated with 635 nm wavelength laser, and (**d**) response and recovery time as a function of excitation wavelength.

**Figure 6:**
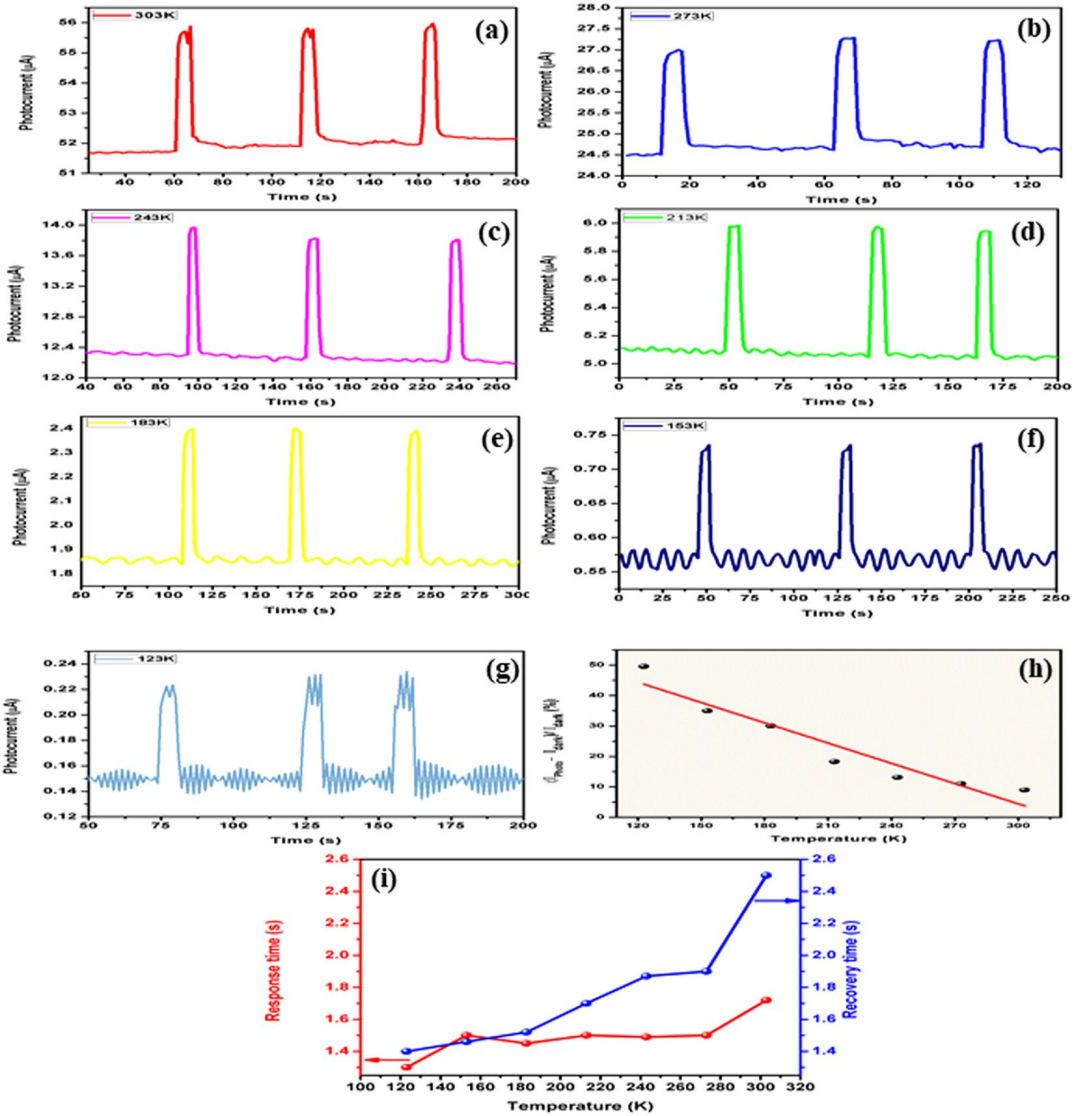
(**a**–**g**) Photoconductive responses (**h**) sensitivity, and (**i**) response and recovery time of rGO based sensor observed at low temperature (303K–123K) of operation.

### Wavelength dependent Sensing Response

Photoresponse in terms of change in current was measured for different excitation wavelengths (λ_exc_ ~ 635, 785, and 1064 nm) while intensity of incident light was kept constant. Figure 5(a) shows a sharp increase in current on exposing light onto the sensor, which quickly returns to dark current as the illumination is turned off. Besides this, the sensor has shown excellent stability and repeatability in response. Maximum photoresponsivity observed at 635 nm laser illumination signify that the transition is interband and occur from the GO region where sp^3^ carbon bonding is dominant (Fig. 5(b)). In Fig. 5(c), response time is calculated from 10% to 90% amplitude of the dark current and recovery time by 90% to 10% amplitude of the dark current. Response and recovery times were found to be 1.5 s and 1.7 s respectively at room temperature for 635 nm laser illumination. Figure 5(d) shows the response and recovery times of the rGO sensor observed for various excitation wavelengths where response and recovery time are found increasing with λ_exc_. Due to involvement of heat generated electron-phonon scattering at higher wavelengths, theoretically the response and recovery time should increase with the rise in temperature and this is also reflected in our experimental data.

### Temperature dependent Sensing Response

Figure 6(a–g) shows the temperature dependent photoconductive response of the sensor in 123K–303K temperature range. During exposure to 635 nm laser, working temperature of sensor is maintained by a temperature controller fitted with the Linkam T95-PE chamber. When the temperature is reduced below 303 K, the sensitivity increases, as shown in Fig. 6(h). The increase in sensitivity is due to less electron scattering with phonons as well as the structural defects in rGO since the phonon density is comparatively less at low temperature. The shift in base line current towards lower value of temperature originates from the standard temperature dependent property of semiconductor.

Figure 6(h) shows the variation of sensitivity of the sensor with respect to operating temperature. In addition, response and recovery time have also been found to be dependent on the operating temperature. As evident from Fig. 6(i), both response and recovery times increase with increase in operating temperature. This behavior can be ascribed to the carrier scattering phenomenon, discussed for sensitivity.

**Figure 7:**
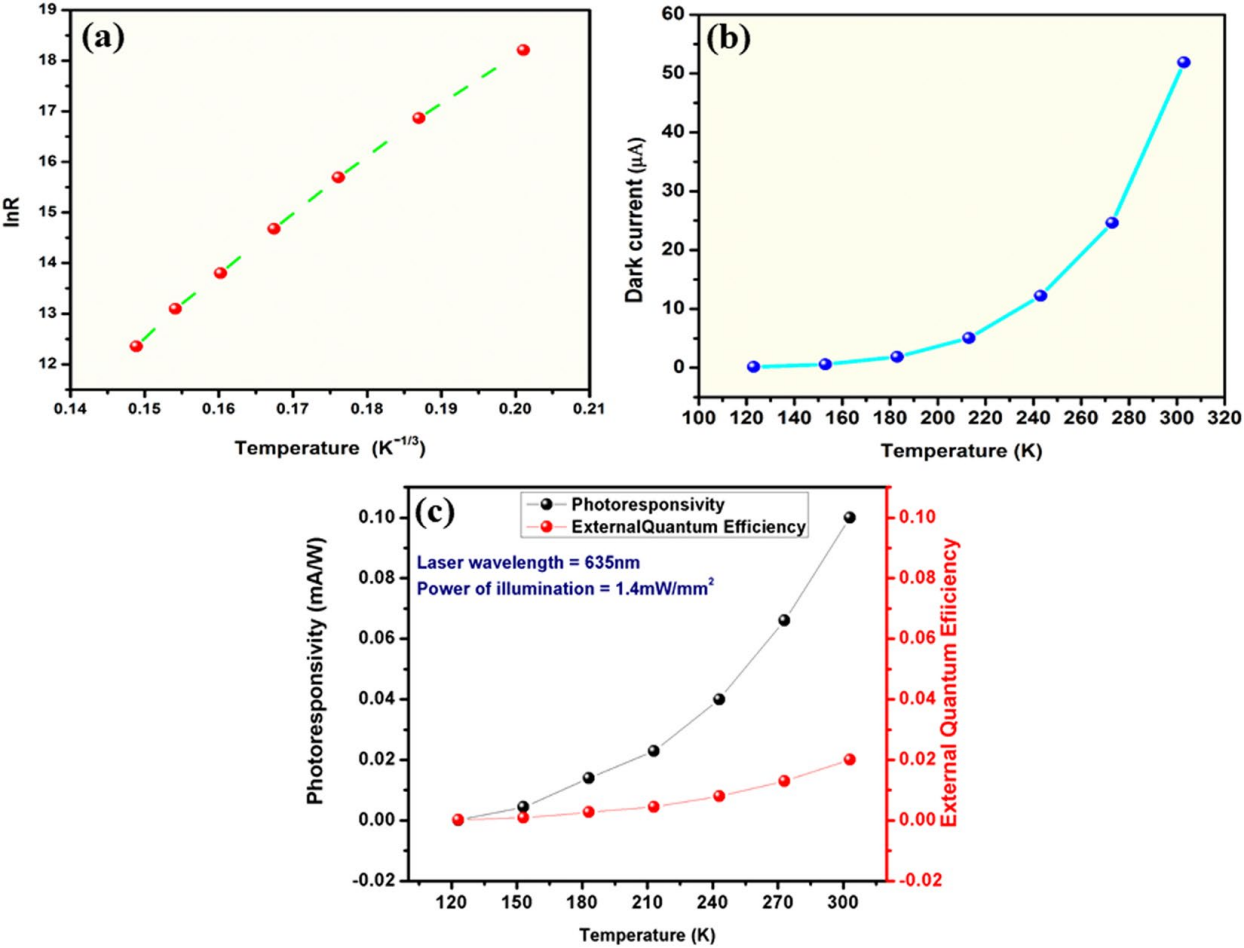
Temperature dependence of (**a**) electrical resistance, (**b**) dark current, and (**c**) photoresponsivity and external quantum efficiency of rGO sensor.

Figure 7(a) shows the temperature dependent electrical resistance where R-T dependence is observed as T^-1/3^ in the temperature region 123K–303K. Such dependence is similar to disordered system where carrier transport is dominated by variable range hopping (VRH) mechanism^49–57^. Each defect site is a charge trapping center and therefore, electron mobility or electron transit time between terminal electrodes heavily depend on the density of defects, which, in turn depends on thermal reduction time from GO to rGO transformation. The experimental results are in close agreement of these arguments are shown in subsequent sections. Figure 7(b) demonstrates that the dark current (refer to Supplementary information file) of sensor increases with rise in operating temperature. Photoresponsivity and external quantum efficiency are directly proportional to the temperature of operation as shown in Fig. 7(c).

**Figure 8:**
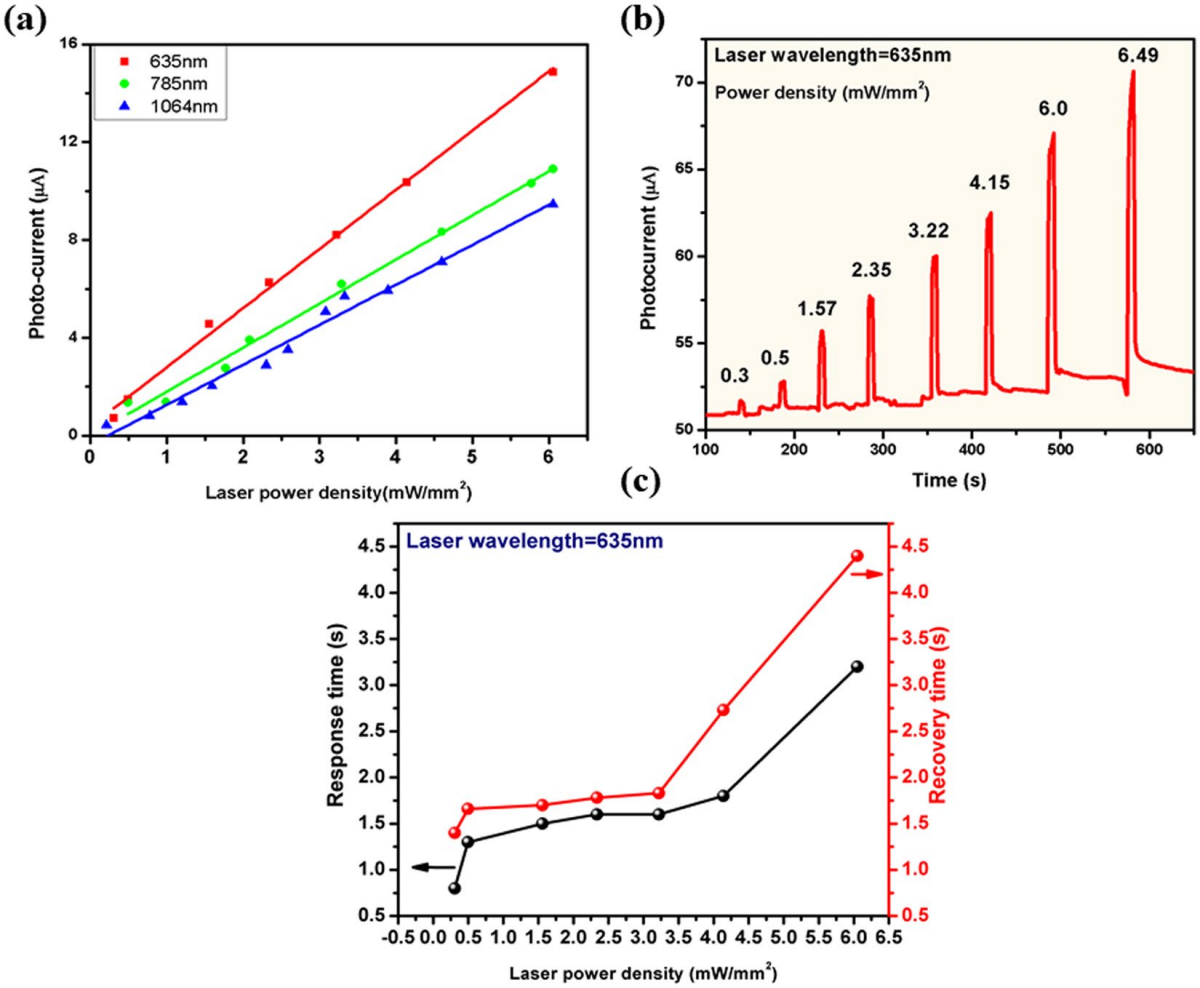
(**a**) Photocurrent as a function of laser power density corresponding to different laser excitation wavelengths, (**b**) Photocurrent response of sensor as a function of laser power density, and (**c**) Response and recovery time behavior at different laser power density.

### Laser power density dependent sensing response

Variation in photo response with increase in laser power density of excitation wavelength (λ_exc_) is depicted in Fig. 8(a). It is observed that the photo-current in the sensor linearly increases with the increase in laser power density of λ_exc_ (Fig. 8(b)). Response and recovery times vary directly with incident laser power as shown in Fig. 8(c). This can be attributed to enhanced carrier scatterings at higher temperatures caused by high power density. This data may be an indicator for safe operation of the sensor device, such that the heating effects of sensor can be avoided.

### Dynamic Sensor response: Sensor Resolution studies

**Figure 9:**
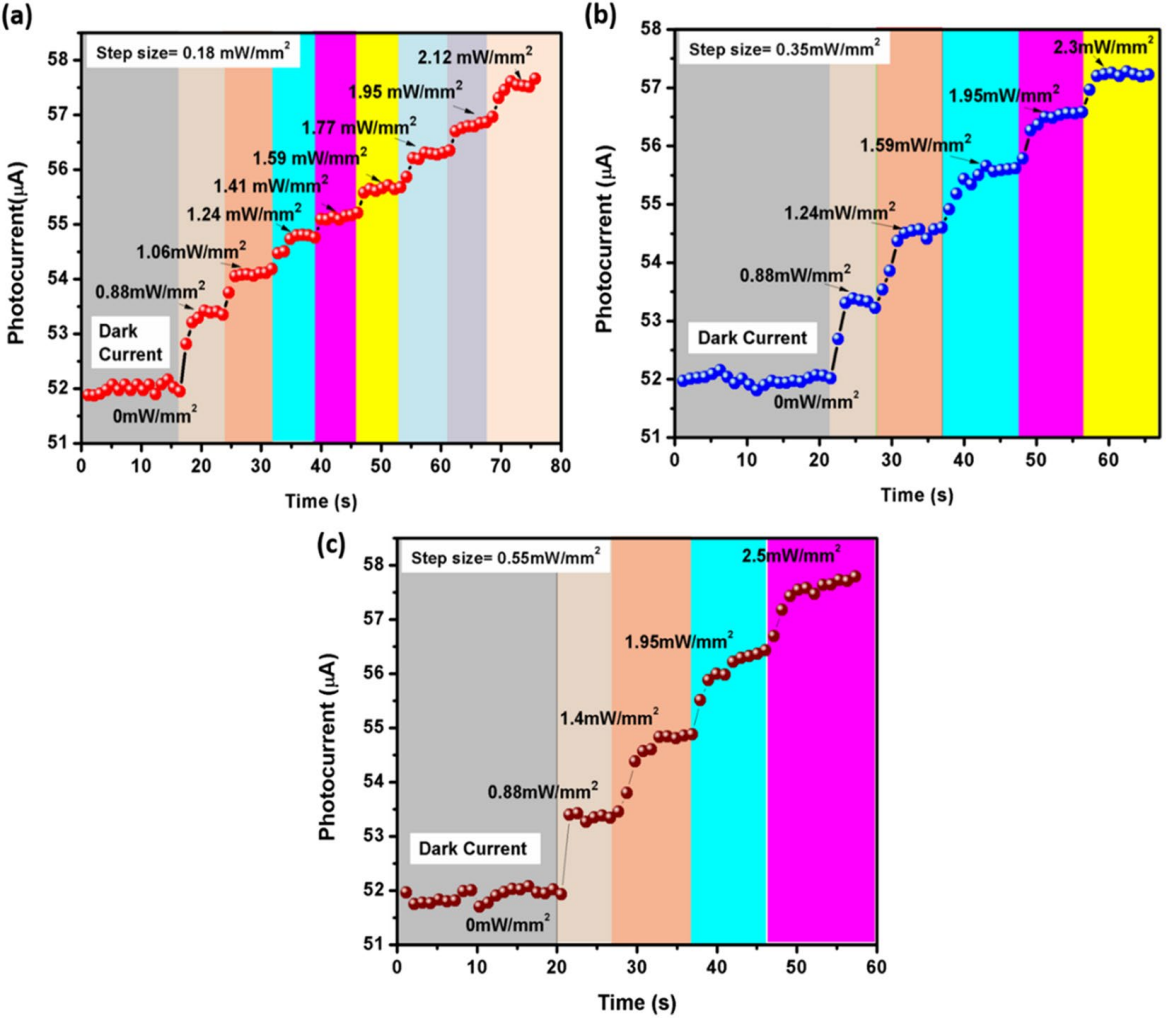
Dynamic photocurrent response curve for λ_exc_ ~ 635 nm with different excitation power density step sizes: (**a**) 0.18 mW/mm^2^, (**b**) 0.35 mW/mm^2^, and (**c**) 0.55 mW/mm^2^.

To ascertain the resolution of the developed sensor, incident power density of 635 nm excitation laser is increased in different step sizes and the obtained photoresponse is shown in Fig. 9. From figure, it is evident that the sensor can detect a minimum of 0.18 mW/mm^2^ change in illumination power density. Although, a large change is observed with this step size, we could not further reduce the step size owing to the limitation of laser power controller.

## Conclusions

In conclusion, reduced graphene oxide (rGO) based self-standing film is produced via modified Hummers’ technique and subsequently annealed at different temperatures for varying time durations. Raman and XRD analysis verified the degree of reduction during transformation from GO to rGO. Optical sensor is developed from the self-standing rGO film. Sensor is exposed to visible to NIR excitations and highest response is observed under 635 nm laser illumination at room temperature. Temperature dependent studies suggest an inverse relation between sensitivity and operating temperature. Highest sensitivity of 49.2% is obtained at 123 K for 635 nm laser with power density of 1.4 mW/mm^2^. Response and recovery times were also found to increase with rise in temperature of operation. Power dependent studies demonstrate linear relation between power-density and sensitivity, and show the safe operation limit beyond which sample heating prolongs the sensor recovery time. Wavelength dependent studies demonstrate that the fabricated rGO sensor is highly sensitive to wide excitation wavelength range, i.e., from visible to NIR. Notably, graphene is compatible with already matured silicon platform for electronics and photonics, making it a potential candidate for economic and large-scale integration into optoelectronic networks and multipixel CMOS read-out circuits. Such rGO based optical sensor performance at low temperature is first report to the best of our knowledge.

## Electronic supplementary material

Figure S(I), Figure S2
