# Supplementary material for: Reduced graphene oxide (rGO) based wideband optical sensor and the role of Temperature, Defect States and Quantum Efficiency
Source: Sci Rep. 2018 Feb 23;8:3537. doi: 10.1038/s41598-018-21686-2 (PMC5824820; doi:10.1038/s41598-018-21686-2)
Supplement: Supplementary file 1 — Figure S(I), Figure S2 [file 41598_2018_21686_MOESM1_ESM.docx]

**Reduced graphene oxide (rGO) based wideband optical sensor and the role of Temperature, Defect States and Quantum Efficiency**

**Abid, Poonam Sehrawat, S.S. Islam*****, Prabhash Mishra & Shahab Ahmad**

Centre for Nanoscience and Nanotechnology,

Jamia Millia Islamia (A Central University), New Delhi-110025, India.

(*) Corresponding Author Email: [sislam@jmi.ac.in](mailto:sislam@jmi.ac.in); Tel.: +91 (11) 26987153.

Supplementary Information

**Terminology and definitions:**

1. **Dark current (*I_Dark_*):**

At any temperature T, the ‘Dark current’ is defined as the current in the sensing circuit without any light illumination, and it is expressed as [1]:

I*_Dark_* =V*_bias_* /R*_T_*, ------------------- (1)

where V*_bias_* is the bias voltage applied across the sensor and R*_T_* is the resistance of the sensor device at temperature T under no illumination.

When T increases, R*_T_* decreases (because of the semiconductor nature of the sensing material), and therefore I*_Dark_* increases. On light illumination of suitable energy or wavelength on the device, excess number of free carriers will generate in the conduction band.

1. **Photoresponsivity (**$\boldsymbol{R}_{\boldsymbol{\lambda}}$**):**

It is defined as the ratio of the intensity of the photo-generated current to that of the incident light at a given wavelength *λ*, and mathematically expressed as [2-4]:

$R_{\lambda}=\frac{\Delta I_{ph}}{P.A}$ ------------------- (2)

Therefore, Photoresponsivity directly depends on the photocurrent, i.e. $R_{\lambda}\alpha$ $\boldsymbol{\Delta}I_{ph}$, where $\boldsymbol{\Delta}I_{ph}$ ($\boldsymbol{\Delta}I_{ph}=$I*_photo_*-I*_Dark_*) is change in photocurrent due to light illumination, P = power density of illuminated light of wavelength *λ*, and A= area of the device. Here P and A are constant.

1. **Sensitivity (S%):**

Sensitivity is given by the ratio of change in current under illumination and dark current [5].

S (%) = $\frac{I_{photo}-I_{Dark}}{I_{Dark}}$ * 100

= $\frac{\boldsymbol{\Delta}I_{ph}}{I_{Dark}}$ * 100 ------------------- (3)

When temperature increases $\boldsymbol{\Delta}I_{ph}$ and I*_Dark_* both will increase. Whether sensitivity will increase/decrease with temperature depends on the value of the ratio ∆I*_ph_* and *I_Dark_.*

A plot showing I*_Dark_*, I*_photo_* and ∆I*_ph_* versus temperature is depicted in fig I.





Figure S1. $I_{Dark},$ $I_{photo}\boldsymbol{,}and\boldsymbol{\Delta}I_{ph}$ versus temperature.

d**) External Quantum Efficiency (EQE%)**:

It is defined as the ratio of electrons flowing out of the device to the photons absorbed by the device, and it is expressed as [2-4].

EQE (%) = $\frac{{1240. R}_{\lambda}}{\lambda(nm)}*100$ ----------------- (4)

It has a direct relationship with photoresponsivity (R*_λ_*) i.e. EQE will increase with increase in photoresponsivity.

The SEM images of the optimized rGO sample at different magnification are shown in Figure S2.


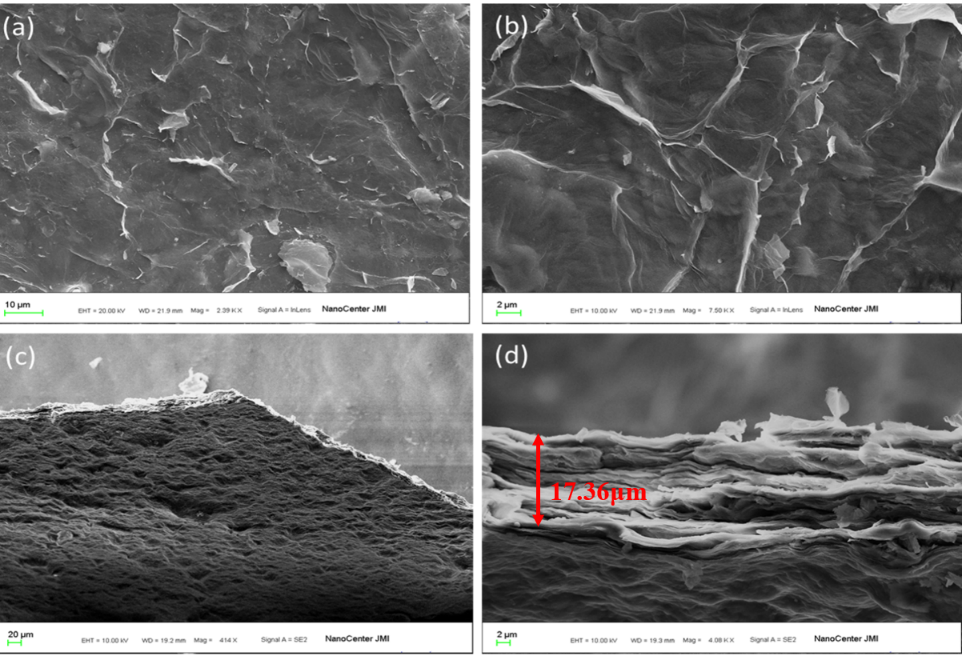


Figure S2 SEM image of self-standing film at (a) low and (b) high magnifications. And cross section views of film at (c) low and (d) high magnifications.

References:

1. Mueller, Thomas, Fengnian Xia, and Phaedon Avouris. "Graphene photodetectors for high-speed optical communications." Nature photonics 4.5 (2010): 297-301.
2. Chitara, B., Panchakarla, L.S., Krupanidhi, S.B. and Rao, C.N.R., 2011. Infrared photodetectors based on reduced graphene oxide and graphene nanoribbons. Advanced Materials, 23(45), pp.5419-5424.
3. Sahatiya, P., Puttapati, S.K., Srikanth, V.V. and Badhulika, S., 2016. Graphene-based wearable temperature sensor and infrared photodetector on a flexible polyimide substrate. Flexible and Printed Electronics, 1(2), p.025006.
4. K. S. Novoselov, A. K. Geim, S. Morozov, D. Jiang, Y. Zhang, S. V. Dubonos, I. V. Grigorieva, and A. A. Firsov, Science, 2004, 306, 666.
5. Sharma, R., Al-Hamry, A., Vijayragavan, S., Benchirouf, A., Sanli, A., Muller, C. and Kanoun, O., 2014, February. Single-wall carbon nanotubes based near-infrared sensors on flexible substrate. In Systems, Signals & Devices (SSD), 2014 11th International Multi-Conference on (pp. 1-5). IEEE.
